# Supplementary material for: Cross-scale drivers of soil fungal diversity in fragmented forests of southwestern China
Source: Commun Biol. 2025 Nov 26;8:1689. doi: 10.1038/s42003-025-09091-8 (PMC12658136; doi:10.1038/s42003-025-09091-8)
Supplement: Supplementary file 2 — Supporting Information [file 42003_2025_9091_MOESM2_ESM.pdf]

Title: Cross-scale drivers of soil fungal diversity in fragmented forests of southwestern China

Yawen Lu[1], Jing Han[1], Shilu Zheng[2], Ying Chen[3*]

Supporting Information

Supplementary Table 1–8

Supplementary Figure 1–11

Supplementary Table 1. The number of sequences and OTUs of each classified fungal functional guild.

	Number of sequences	Number of OTUs
Animal parasite	13199	168
Ectomycorrhizal	144777	263
Foliar endophyte	2779	39
Litter saprotroph	25497	379
Mycoparasite	15165	77
Plant pathogen	28258	323
Soil saprotroph	90988	518
Unspecified saprotroph	22059	243
Wood saprotroph	21932	375

Supplementary Table 2. Spearman rank correlation between richness, Shannon diversity, composition abundance of different fungal functional guilds and local-, patch- and landscape-scale factors. Local factors include soil PC1, tree composition, tree richness, elevation and slope. Patch factors include $\log_{10}$ transformed patch size and edge distance. Landscape factors include $\log_{10}$ transformed mean of patch area (mean area), and patch richness. Shown were the correlation strength r-value and significance *p*-value. The significant correlations were marked in bold.

**Functional guild**	**Environmental variables**	**(a) Richness**		**(b) Shannon**		**(c) Composition**		**(d) Abundance**	
		r	*p*	r	*p*	r	*p*	r	*p*
Ectomycorrhizal	Soil PC1	**-0.480**	**0.008**	**0.543**	**0.003**	**0.632**	**<0.001**	**-0.630**	**<0.001**
Ectomycorrhizal	Tree composition	**0.770**	**<0.001**	-0.365	0.053	**-0.785**	**<0.001**	**0.750**	**<0.001**
Ectomycorrhizal	Tree richness	0.077	0.692	-0.332	0.078	-0.084	0.665	0.235	0.221
Ectomycorrhizal	Elevation	0.193	0.316	0.175	0.361	-0.118	0.542	0.068	0.725
Ectomycorrhizal	Slope	-0.275	0.149	-0.035	0.858	0.234	0.222	-0.161	0.404
Ectomycorrhizal	Patch size	0.028	0.886	-0.048	0.803	-0.009	0.962	0.139	0.472
Ectomycorrhizal	Edge distance	0.056	0.774	0.023	0.907	-0.146	0.450	0.085	0.663
Ectomycorrhizal	Mean area	**0.373**	**0.046**	-0.024	0.903	-0.307	0.105	**0.406**	**0.029**
Ectomycorrhizal	Patch richness	-0.135	0.484	0.024	0.901	0.141	0.465	-0.173	0.371
Foliar endophyte	Soil PC1	0.266	0.164	**0.370**	**0.049**	0.118	0.542	0.216	0.261
Foliar endophyte	Tree composition	**-0.661**	**<0.001**	**-0.571**	**0.001**	**-0.551**	**0.002**	**-0.539**	**0.003**
Foliar endophyte	Tree richness	0.256	0.180	0.016	0.934	0.338	0.072	0.263	0.169
Foliar endophyte	Elevation	-0.347	0.065	0.219	0.253	**-0.462**	**0.012**	-0.320	0.090
Foliar endophyte	Slope	0.080	0.678	0.193	0.316	0.192	0.319	-0.033	0.864
Foliar endophyte	Patch size	-0.080	0.682	0.115	0.553	-0.034	0.862	-0.049	0.800
Foliar endophyte	Edge distance	-0.007	0.970	0.272	0.153	-0.015	0.939	-0.055	0.777
Foliar endophyte	Mean area	**-0.540**	**0.003**	-0.136	0.480	**-0.519**	**0.004**	**-0.583**	**0.001**
Foliar endophyte	Patch richness	0.099	0.608	-0.281	0.140	0.257	0.178	0.120	0.536
Plant pathogen	Soil PC1	**0.564**	**0.001**	**0.402**	**0.032**	**0.572**	**0.001**	**0.500**	**0.006**
Plant pathogen	Tree composition	**-0.835**	**<0.001**	**-0.515**	**0.005**	**-0.875**	**<0.001**	**-0.721**	**<0.001**
Plant pathogen	Tree richness	0.082	0.671	0.206	0.283	-0.053	0.784	-0.103	0.594
Plant pathogen	Elevation	-0.105	0.587	-0.074	0.700	-0.355	0.059	-0.272	0.152
Plant pathogen	Slope	0.262	0.169	0.314	0.097	**0.453**	**0.014**	0.149	0.442
Plant pathogen	Patch size	0.067	0.732	-0.063	0.745	-0.095	0.625	-0.098	0.612
Plant pathogen	Edge distance	-0.073	0.708	-0.246	0.198	-0.217	0.258	-0.145	0.452
Plant pathogen	Mean area	-0.252	0.187	-0.163	0.397	**-0.454**	**0.013**	**-0.418**	**0.024**
Plant pathogen	Patch richness	0.093	0.633	0.331	0.080	0.221	0.250	0.110	0.570
Animal parasite	Soil PC1	**0.452**	**0.014**	0.195	0.309	**0.582**	**0.001**	**0.637**	**<0.001**
Animal parasite	Tree composition	**-0.742**	**<0.001**	**-0.621**	**<0.001**	**-0.874**	**<0.001**	**-0.751**	**<0.001**
Animal parasite	Tree richness	0.171	0.375	0.258	0.176	-0.063	0.745	0.001	0.997
Animal parasite	Elevation	-0.043	0.824	-0.090	0.641	-0.253	0.185	-0.045	0.816
Animal parasite	Slope	0.352	0.061	0.322	0.088	0.313	0.098	0.257	0.179
Animal parasite	Patch size	0.088	0.648	0.087	0.653	-0.032	0.870	-0.019	0.923
Animal parasite	Edge distance	-0.127	0.510	-0.222	0.247	-0.171	0.374	-0.084	0.665

Animal parasite	Mean area	-0.273	0.152	-0.148	0.445	**-0.476**	**0.009**	**-0.404**	**0.030**
Animal parasite	Patch richness	0.024	0.903	-0.043	0.824	0.225	0.242	0.030	0.878
Mycoparasite	Soil PC1	**0.445**	**0.016**	**0.694**	**<0.001**	**0.680**	**<0.001**	**-0.442**	**0.017**
Mycoparasite	Tree composition	**-0.720**	**<0.001**	**-0.712**	**<0.001**	**-0.806**	**<0.001**	**0.458**	**0.013**
Mycoparasite	Tree richness	-0.074	0.701	-0.276	0.147	-0.061	0.752	0.180	0.350
Mycoparasite	Elevation	0.063	0.744	0.112	0.560	0.118	0.542	0.187	0.331
Mycoparasite	Slope	0.191	0.322	**0.386**	**0.038**	0.342	0.069	-0.222	0.247
Mycoparasite	Patch size	0.111	0.567	0.224	0.243	0.189	0.327	-0.007	0.969
Mycoparasite	Edge distance	-0.101	0.603	-0.010	0.958	0.073	0.706	0.012	0.951
Mycoparasite	Mean area	-0.056	0.773	-0.183	0.341	-0.175	0.363	0.269	0.159
Mycoparasite	Patch richness	0.040	0.836	-0.029	0.882	-0.009	0.965	0.056	0.774
Wood saprotroph	Soil PC1	**0.514**	**0.004**	0.326	0.085	**0.424**	**0.023**	**0.583**	**0.001**
Wood saprotroph	Tree composition	**-0.807**	**<0.001**	**-0.684**	**<0.001**	**-0.788**	**<0.001**	**-0.564**	**0.002**
Wood saprotroph	Tree richness	0.039	0.842	0.311	0.100	0.146	0.450	-0.296	0.120
Wood saprotroph	Elevation	-0.173	0.368	-0.171	0.374	-0.350	0.063	0.027	0.889
Wood saprotroph	Slope	0.261	0.171	0.228	0.233	**0.369**	**0.049**	0.068	0.726
Wood saprotroph	Patch size	-0.055	0.777	-0.005	0.981	-0.044	0.819	-0.030	0.878
Wood saprotroph	Edge distance	-0.190	0.325	-0.058	0.766	-0.306	0.106	-0.099	0.611
Wood saprotroph	Mean area	-0.332	0.078	-0.250	0.192	**-0.394**	**0.034**	-0.252	0.188
Wood saprotroph	Patch richness	0.102	0.599	0.030	0.879	0.154	0.425	0.014	0.942
Litter saprotroph	Soil PC1	**0.445**	**0.016**	**0.387**	**0.039**	**0.667**	**<0.001**	**0.398**	**0.033**
Litter saprotroph	Tree composition	**-0.746**	**<0.001**	**-0.593**	**0.001**	**-0.893**	**<0.001**	**-0.523**	**0.004**
Litter saprotroph	Tree richness	0.269	0.158	0.230	0.229	-0.168	0.384	0.146	0.451
Litter saprotroph	Elevation	**-0.371**	**0.048**	-0.052	0.788	-0.154	0.423	-0.198	0.302
Litter saprotroph	Slope	0.292	0.124	0.212	0.270	0.340	0.071	0.154	0.425
Litter saprotroph	Patch size	-0.262	0.170	0.297	0.118	0.105	0.589	-0.321	0.090
Litter saprotroph	Edge distance	-0.365	0.052	0.134	0.489	-0.065	0.738	-0.303	0.110
Litter saprotroph	Mean area	**-0.427**	**0.021**	-0.287	0.132	-0.339	0.072	-0.349	0.064
Litter saprotroph	Patch richness	0.037	0.849	0.048	0.806	0.051	0.794	-0.086	0.659
Soil saprotroph	Soil PC1	0.262	0.170	0.281	0.139	**-0.566**	**0.002**	0.068	0.725
Soil saprotroph	Tree composition	**-0.391**	**0.036**	**-0.624**	**<0.001**	**0.623**	**<0.001**	0.217	0.256
Soil saprotroph	Tree richness	**0.421**	**0.023**	0.166	0.391	0.299	0.114	0.279	0.142
Soil saprotroph	Elevation	0.103	0.597	-0.176	0.359	-0.076	0.695	0.360	0.056
Soil saprotroph	Slope	-0.065	0.736	0.357	0.057	**-0.567**	**0.001**	-0.146	0.450
Soil saprotroph	Patch size	-0.087	0.654	0.126	0.516	-0.065	0.738	-0.149	0.439
Soil saprotroph	Edge distance	-0.067	0.730	0.066	0.734	0.178	0.355	0.015	0.937
Soil saprotroph	Mean area	-0.058	0.763	-0.174	0.367	-0.005	0.979	0.077	0.693
Soil saprotroph	Patch richness	-0.128	0.508	0.100	0.606	0.199	0.301	-0.288	0.130
Unspecified saprotroph	Soil PC1	0.295	0.120	0.128	0.506	**-0.609**	**0.001**	0.291	0.126
Unspecified saprotroph	Tree composition	**-0.503**	**0.005**	-0.353	0.061	**0.870**	**<0.001**	-0.366	0.051
Unspecified saprotroph	Tree richness	0.043	0.826	-0.003	0.988	0.077	0.691	-0.161	0.403
Unspecified saprotroph	Elevation	0.013	0.948	**-0.391**	**0.037**	-0.067	0.729	0.285	0.134
Unspecified saprotroph	Slope	0.192	0.319	0.260	0.173	-0.266	0.164	0.061	0.755
Unspecified saprotroph	Patch size	0.243	0.204	-0.298	0.116	-0.176	0.362	**0.423**	**0.022**
Unspecified saprotroph	Edge distance	0.129	0.506	-0.047	0.807	0.044	0.822	0.225	0.240
Unspecified saprotroph	Mean area	**-0.419**	**0.024**	-0.297	0.118	0.290	0.127	-0.277	0.146
Unspecified saprotroph	Patch richness	0.287	0.131	0.284	0.136	-0.097	0.618	0.172	0.373

Supplementary Table 3. R^2 and *p*-values for the correlation between the diversity indices of each fungal functional guild of and the two major Principal Coordinates Analysis (PCoA) axes of tree composition.

Functional guilds	Diversity	r^2	*p*-value
Ectomycorrhizal	Richness	**0.638**	**0.004**
	Shannon	**0.307**	**0.028**
	Composition	**0.694**	**0.004**
	Abundance	**0.485**	**0.004**
Foliar endophyte	Richness	**0.490**	**0.004**
	Shannon	**0.330**	**0.016**
	Composition	**0.576**	**0.004**
	Abundance	**0.329**	**0.016**
Plant pathogen	Richness	**0.820**	**0.004**
	Shannon	**0.309**	**0.016**
	Composition	**0.904**	**0.004**
	Abundance	**0.437**	**0.016**
Animal parasite	Richness	**0.637**	**0.004**
	Shannon	**0.452**	**0.004**
	Composition	**0.882**	**0.004**
	Abundance	**0.626**	**0.004**
Mycoparasite	Richness	**0.519**	**0.004**
	Shannon	**0.616**	**0.004**
	Composition	**0.735**	**0.004**
	Abundance	0.172	0.356
Wood saprotroph	Richness	**0.689**	**0.004**
	Shannon	**0.430**	**0.004**
	Composition	**0.834**	**0.004**
	Abundance	0.001	1
Litter saprotroph	Richness	**0.814**	**0.004**
	Shannon	**0.339**	**0.008**
	Composition	**0.869**	**0.004**
	Abundance	0.229	0.124
Soil saprotroph	Richness	**0.317**	**0.04**
	Shannon	**0.386**	**0.008**
	Composition	**0.584**	**0.004**
	Abundance	0.051	1
Unspecified saprotroph	Richness	**0.303**	**0.04**
	Shannon	0.141	0.468
	Composition	**0.764**	**0.004**
	Abundance	0.149	0.448

Supplementary Table 4. The best model predicting the diversity and community composition of different fungal functional guilds based on LMM. Shown were the response variables, best predictors (combination), estimated slopes, standard errors (se), t-values (t) and *p*-values (*p*), AIC_c and marginal R^2. Background colors indicate different diversity indices of richness, Shannon diversity, community composition, and abundance.

Response variable	Best predictors	Estimate	se	t-value	*p*-value	AIC_c	marginal R^2
Animal parasite richness	Tree composition	0.722	0.128	5.651	<0.001	75.42	0.53
Ectomycorrhizal richness	Tree composition	0.726	0.122	5.933	<0.001	70.81	0.57
Foliar endophyte richness	Tree composition	0.606	0.143	4.233	<0.001	80.06	0.38
Litter saprotroph richness	Tree composition	0.737	0.111	6.632	<0.001	67.11	0.65
	Tree richness	0.340	0.103	3.317	0.004		
Mycoparasite richness	Tree composition	0.728	0.141	5.154	<0.001	76.77	0.51
Plant pathogen richness	Tree composition	0.844	0.098	8.615	<0.001	61.06	0.73
Soil saprotroph richness	Soil PC1	0.449	0.171	2.629	0.014	91.73	0.29
	Tree richness	0.470	0.168	2.795	0.010		
Unspecified saprotroph richness	Tree composition	0.506	0.162	3.125	0.005	87.32	0.26
Wood saprotroph richness	Tree composition	0.795	0.115	6.890	<0.001	69.65	0.63
Animal parasite Shannon	Tree composition	0.668	0.157	4.252	<0.001	82.48	0.46
	Tree richness	0.458	0.134	3.420	0.003		
Ectomycorrhizal Shannon	Soil PC1	0.483	0.185	2.603	0.016	89.27	0.22
Foliar endophyte Shannon	Tree composition	0.561	0.162	3.460	0.002	87.38	0.30
Litter saprotroph Shannon	Patch size	0.517	0.200	2.583	0.024	85.00	0.53
	Tree composition	0.505	0.133	3.787	0.001		
	Tree richness	0.463	0.114	4.049	0.001		
Mycoparasite Shannon	Tree composition	0.760	0.138	5.524	<0.001	71.77	0.53
Plant pathogen Shannon	Patch richness	0.540	0.137	3.930	0.001	84.30	0.59
	Tree composition	0.627	0.142	4.412	<0.001		
	Tree richness	0.402	0.137	2.933	0.007		
Soil saprotroph Shannon	Tree composition	0.628	0.151	4.146	<0.001	84.58	0.38
Unspecified saprotroph Shannon	~1 (null model)	-	-	-	-	93.39	-
Wood saprotroph Shannon	Tree composition	0.737	0.140	5.269	<0.001	76.91	0.59
	Tree richness	0.553	0.124	4.443	<0.001		
Animal parasite composition	mean area	0.230	0.064	3.622	0.001	42.66	0.89
	Tree composition	0.853	0.064	13.404	<0.001		
Ectomycorrhizal composition	Tree composition	0.813	0.107	7.629	<0.001	65.64	0.68
Foliar endophyte composition	Tree composition	0.561	0.161	3.473	0.002	79.86	0.30
Litter saprotroph composition	Tree composition	0.913	0.072	12.600	<0.001	43.12	0.86
Mycoparasite composition	Elevation	0.294	0.110	2.681	0.013	59.38	0.77
	Tree composition	0.967	0.088	10.974	<0.001		
Plant pathogen composition	mean area	0.243	0.064	3.780	0.002	37.56	0.90
	Tree composition	0.860	0.060	14.279	<0.001		
Soil saprotroph composition	Soil PC1	0.464	0.128	3.629	0.001	73.72	0.71
	Tree composition	0.364	0.139	2.625	0.015		
	Slope	0.303	0.116	2.606	0.015		

Unspecified saprotroph composition	Tree composition	0.834	0.098	8.492	<0.001	53.31	0.73
Wood saprotroph composition	Tree composition	0.932	0.089	10.509	<0.001	58.96	0.77
	Tree richness	0.341	0.076	4.483	0.001		
Animal parasite abundance	Tree composition	0.715	0.134	5.345	<0.001	74.78	0.53
Ectomycorrhizal abundance	Tree composition	0.640	0.122	5.226	<0.001	71.75	0.49
Foliar endophyte abundance	mean area	0.593	0.220	2.693	0.015	85.76	0.29
Litter saprotroph abundance	~1 (null model)	-	-	-	-	83.91	-
Mycoparasite abundance	Tree composition	0.387	0.172	2.250	0.033	91.48	0.15
Plant pathogen abundance	Tree composition	0.577	0.153	3.782	0.001	82.46	0.35
Soil saprotroph abundance	Elevation	0.547	0.177	3.082	0.006	89.06	0.27
Unspecified saprotroph abundance	Patch size	0.405	0.173	2.339	0.027	91.84	0.16
Wood saprotroph abundance	~1 (null model)	-	-	-	-	78.22	-

Supplementary Table 5. Richness and relative abundance of ectomycorrhizal (ECM) host tree species in each plot across three forest types (Lowland, Montane, and Limestone) in Xishuangbanna.

**Plot**	**Forest type**	**ECM host richness**	**ECM host relative abundance**
1	Lowland	0	0.00
2	Montane	4	0.17
3	Lowland	0	0.00
4	Montane	4	0.19
5	Montane	6	0.24
6	Lowland	2	0.02
7	Lowland	4	0.08
8	Lowland	0	0.00
9	Lowland	0	0.00
10	Montane	4	0.10
11	Lowland	2	0.07
12	Montane	3	0.21
13	Limestone	0	0.00
14	Lowland	1	0.05
15	Montane	4	0.33
16	Limestone	0	0.00
17	Limestone	0	0.00
18	Montane	3	0.04
19	Limestone	0	0.00
20	Limestone	0	0.00
21	Montane	4	0.14
22	Lowland	4	0.09
23	Limestone	1	0.01
24	Limestone	1	0.01
25	Lowland	1	0.02
26	Montane	3	0.32
27	Montane	3	0.25
28	Limestone	0	0.00
29	Lowland	0	0.00
30	Montane	4	0.35

Supplementary Table 6. Summary of the 30 sampling plots within 17 fragmented forest patches in Xishuangbanna, Yunnan Province, China. Information includes the number of subplots, forest type, patch size, and edge distance for each plot.

Fragmented Patch	Plot	No. subplot	Forest type	Patch size (ha)	Edge distance (m)
**1**	1	12	Lowland	1.71	10
**2**	2	6	Montane	4.86	10
**3**	3	8	Lowland	6.3	20
**4**	4	8	Montane	20.7	10
**5**	5	7	Montane	54.9	309.8
**6**	6	15	Lowland	65.16	200
	7	6	Lowland	65.16	30
	8	8	Lowland	65.16	30
**7**	9	6	Lowland	76.14	124
**8**	10	6	Montane	87.57	20
**9**	11	8	Lowland	100.1074	100
	12	6	Montane	100.1074	20
**10**	13	7	Limestone	167.85	50
**11**	14	7	Lowland	329.85	517.4
	15	6	Montane	329.85	313
**12**	16	9	Limestone	518.31	200
	17	5	Limestone	518.31	342.3
**13**	18	9	Montane	925.83	50
**14**	19	6	Limestone	995.4	50
	20	8	Limestone	995.4	300
**15**	21	7	Montane	1748.25	100
**16**	22	7	Lowland	3283.02	469.6
	23	11	Limestone	3283.02	200
	24	8	Limestone	3283.02	485
**17**	25	8	Lowland	13872.87	1047
	26	5	Montane	13872.87	427
	27	7	Montane	13872.87	935
	28	10	Limestone	13872.87	254
	29	14	Lowland	13872.87	10
	30	7	Montane	13872.87	185.6

Supplementary Table 7. The strength (r-value, lower left corner) and significance (*p*-value, top right corner) of Spearman rank correlation between all the soil properties. Soil properties include pH, nitrogen (N), organic matter (OM), phosphorus (P), potassium (K) and calcium (Ca) content.

	pH	N	OM	P	K	Ca
pH		<0.001	0.030	<0.001	0.363	<0.001
N	0.594		<0.001	<0.001	0.158	<0.001
OM	0.402	0.913		0.001	0.025	0.002
P	0.743	0.814	0.584		0.303	<0.001
K	-0.175	-0.269	-0.415	-0.198		0.223
Ca	0.907	0.697	0.548	0.814	-0.233	

Supplementary Table 8. Spatial autocorrelation of predicted soil fungal diversity based on multivariate linear mixed-effects models (LMM). Moran's I values and corresponding *p*-values are reported for the richness, Shannon diversity, abundance, and community composition of the nine fungal functional guilds.

Diversity indices	Moran's I	*p*-value
Animal parasite richness	-0.098	0.840
Ectomycorrhizal richness	-0.037	0.506
Foliar endophyte richness	-0.094	0.824
Litter saprotroph richness	-0.053	0.609
Mycoparasite richness	-0.077	0.743
Plant pathogen richness	-0.047	0.572
Soil saprotroph richness	-0.092	0.821
Unspecified saprotroph richness	-0.122	0.916
Wood saprotroph richness	-0.106	0.871
Animal parasite Shannon	-0.046	0.566
Ectomycorrhizal Shannon	-0.091	0.809
Foliar endophyte Shannon	-0.076	0.743
Litter saprotroph Shannon	-0.098	0.840
Mycoparasite Shannon	-0.070	0.713
Plant pathogen Shannon	-0.082	0.771
Soil saprotroph Shannon	-0.088	0.799
Unspecified saprotroph Shannon	-0.062	0.685
Wood saprotroph Shannon	-0.046	0.566
Animal parasite abundance	-0.089	0.804
Ectomycorrhizal abundance	-0.101	0.856
Foliar endophyte abundance	-0.082	0.777
Litter saprotroph abundance	0.009	0.232
Mycoparasite abundance	-0.081	0.780
Plant pathogen abundance	-0.054	0.619
Soil saprotroph abundance	-0.102	0.857
Unspecified saprotroph abundance	-0.096	0.832
Wood saprotroph abundance	-0.062	0.663
Animal parasite composition	-0.081	0.762
Ectomycorrhizal composition	-0.080	0.759
Foliar endophyte composition	-0.101	0.860
Litter saprotroph composition	-0.108	0.884
Mycoparasite composition	-0.099	0.844
Plant pathogen composition	-0.052	0.606
Soil saprotroph composition	-0.112	0.891
Unspecified saprotroph composition	-0.073	0.724
Wood saprotroph composition	-0.100	0.858

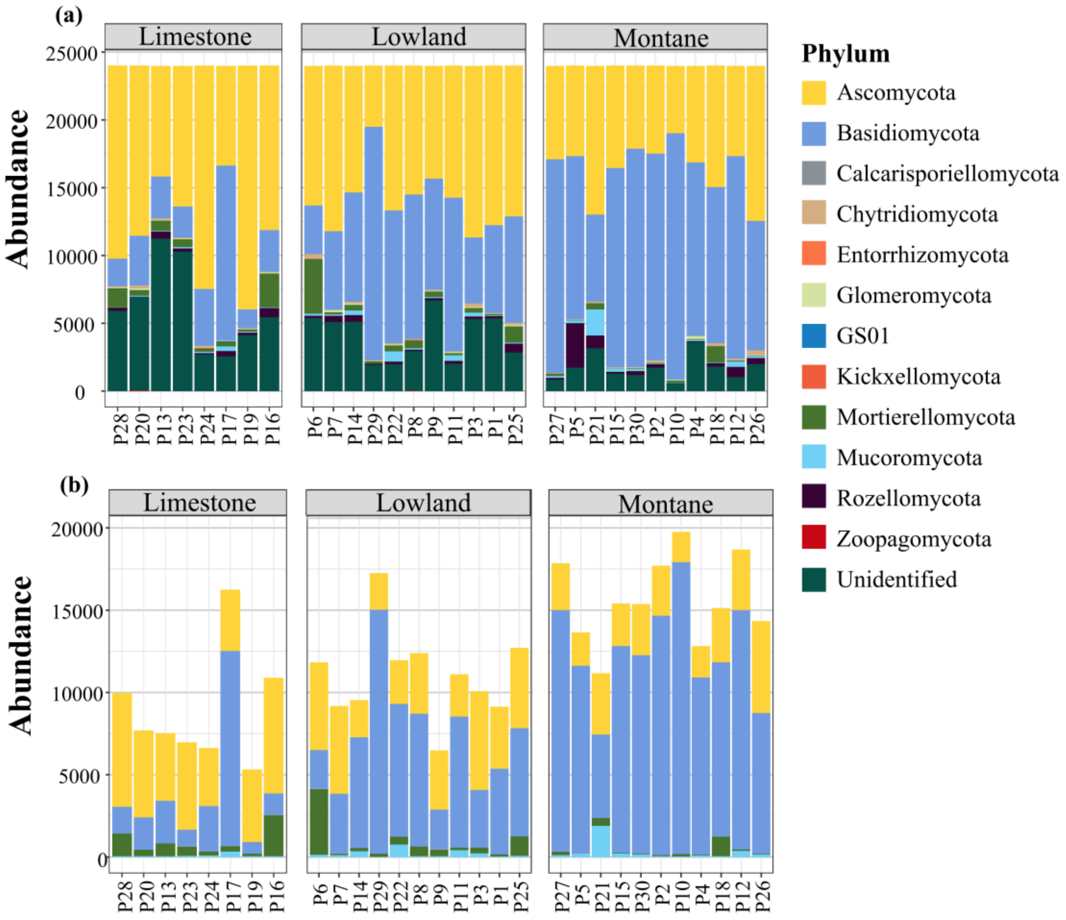

Supplementary Figure 1. Soil fungal abundance based on rarefied sequence counts across three forest types. (a) Relative abundance of all fungal phyla and (b) represented fungal functional guilds from 30 sampling plots in limestone, lowland, and montane forests. Colors indicate different fungal phyla as shown in the legend.

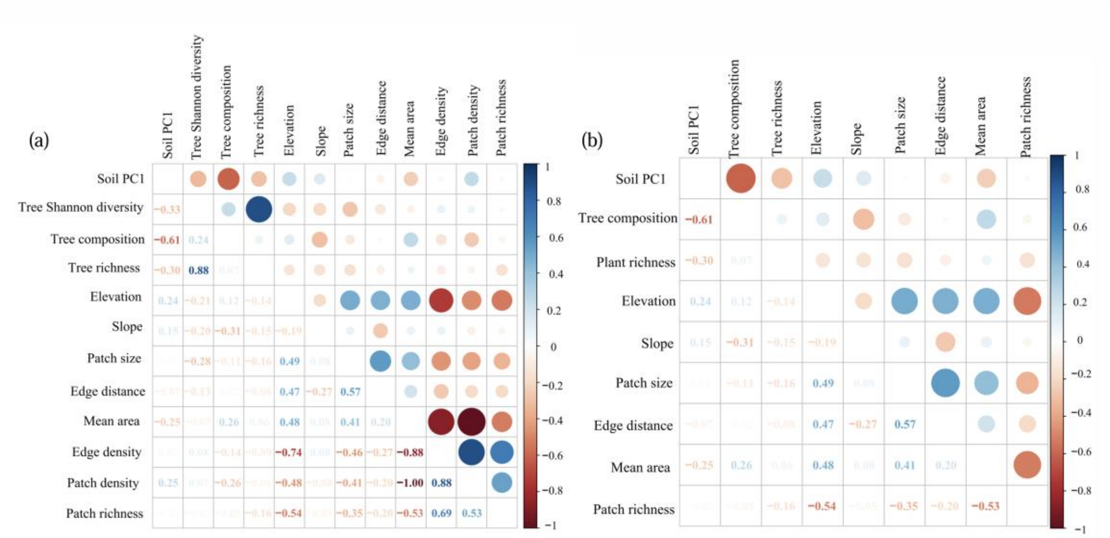

Supplementary Figure 2. Spearman rank correlation between continuous environmental variables. (a) Correlation matrix before and (b) after removing variables with high collinearity ($r \geq 0.7$). The local variables include soil PC1, tree Shannon diversity, tree composition, tree richness, elevation and slope. The patch variables include $\log_{10}$ transformed patch size, edge distance, and the landscape variables include $\log_{10}$ transformed mean of patch area (mean area), edge density, patch density, and patch richness.

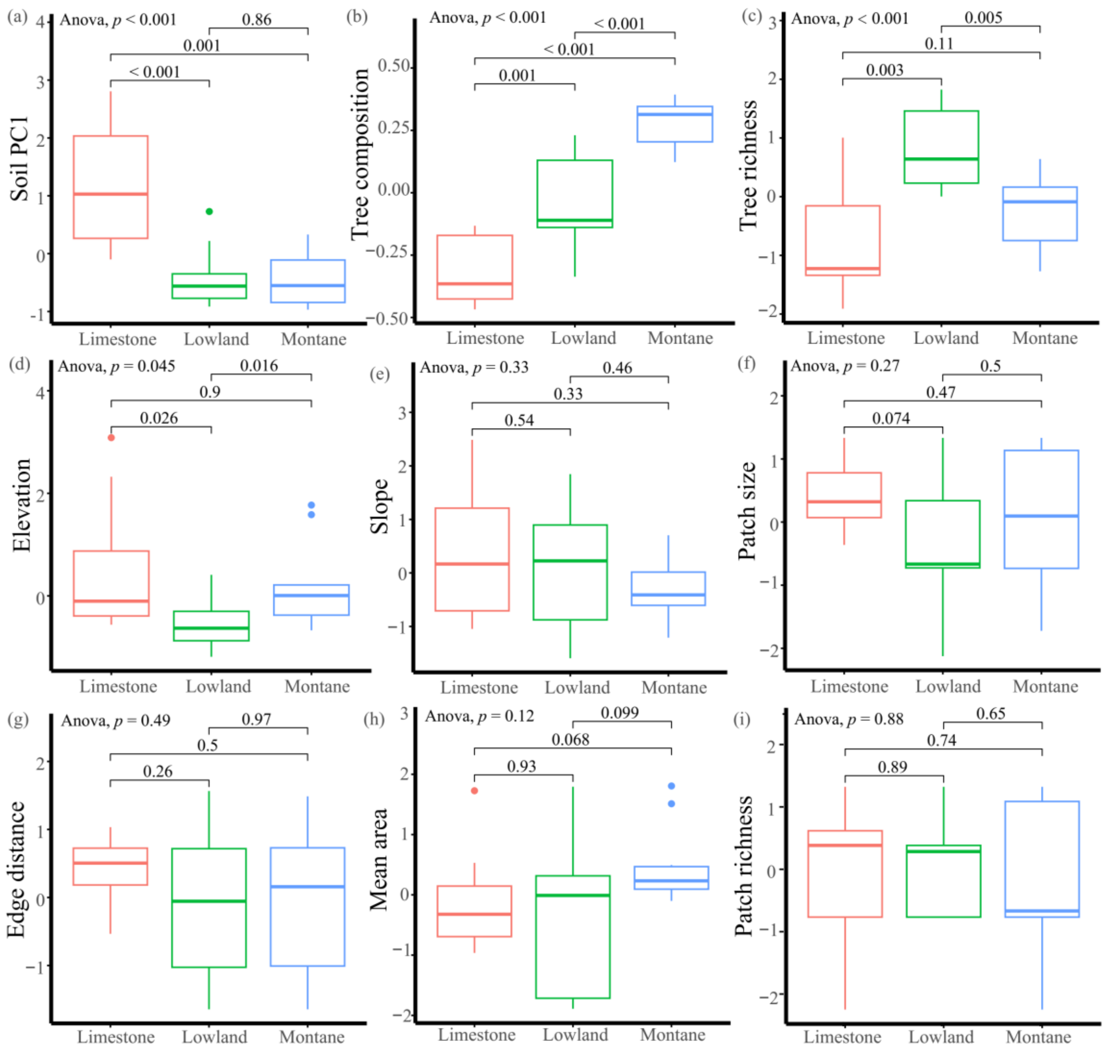

Supplementary Figure 3. Variation in environmental factors across three forest types. Variation in local (a)–(e), patch (f)–(g), and landscape (h)–(i) scale environmental factors across limestone, lowland, and montane forests (n = 30 biologically independent samples). Local factors include soil PC1, tree composition, tree richness, elevation and slope. Patch factors include $\log_{10}$ transformed patch size and edge distance. Landscape factors include $\log_{10}$ transformed mean of patch area (mean area), and patch richness. The significance between forest types for each environmental factor was tested by *p*-value with TukeyHSD test.

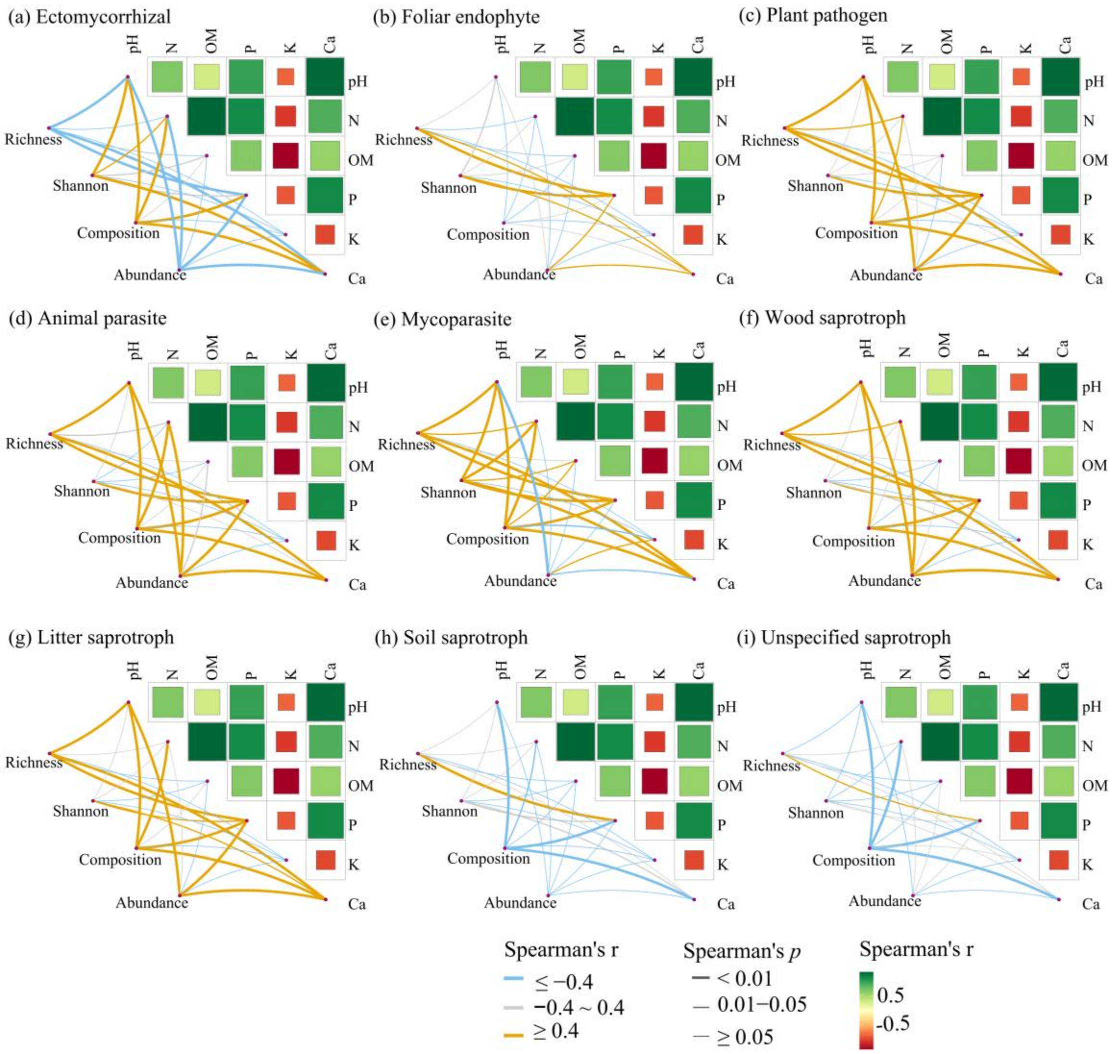

Supplementary Figure 4. Spearman rank correlations between soil properties and fungal functional guild diversity. (a)–(i) The correlations between soil properties and the richness, Shannon diversity, community composition, and abundance of nine fungal functional guilds. Different colors of the lines indicate the Spearman rank correlation between the fungal diversity index and soil properties, and the thickness indicates the correlation significance. The upper right corner is the Spearman rank correlation among soil properties. Soil properties include pH, nitrogen (N), organic matter (OM), phosphorus (P), potassium (K) and calcium (Ca) content.

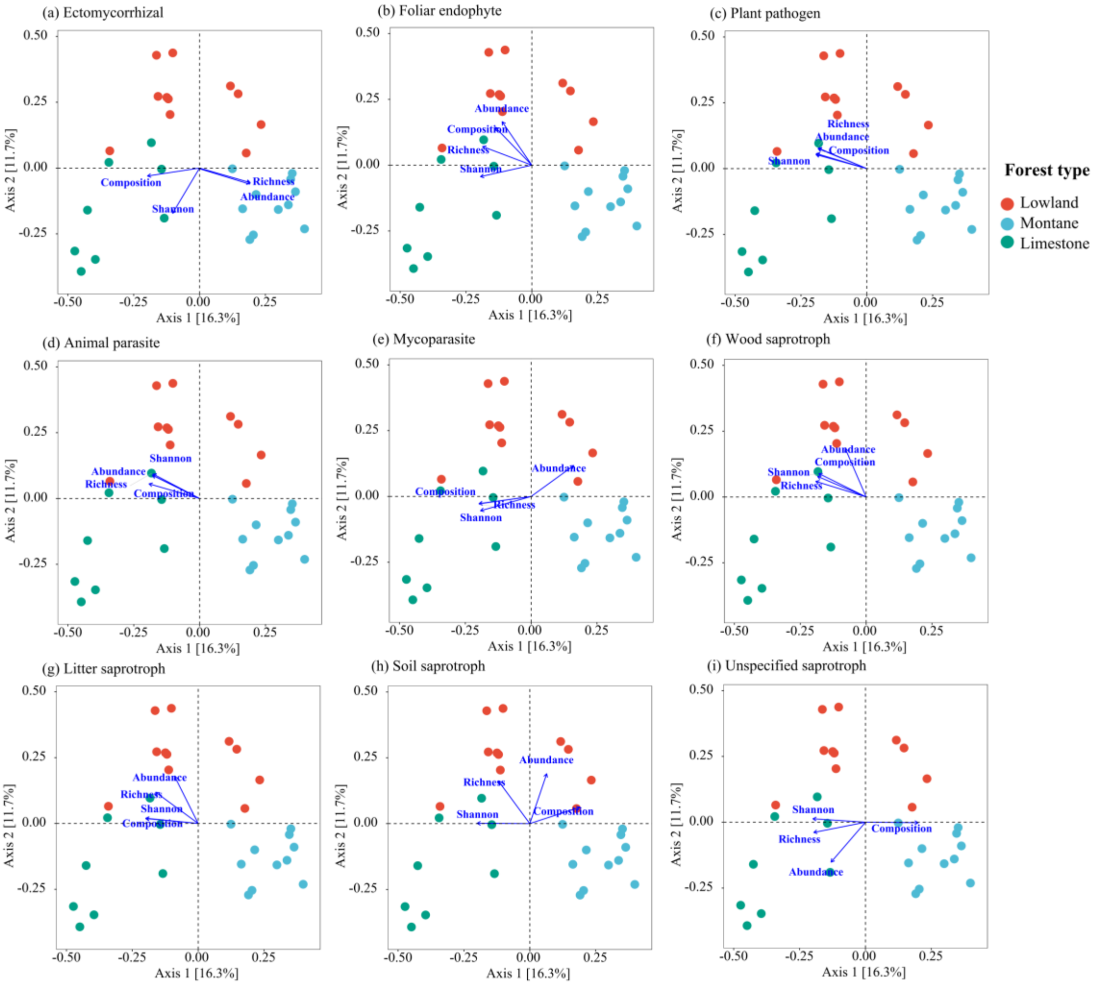

Supplementary Figure 5. Variation in tree composition across forest types and its association with fungal functional guilds. (a)–(i) Principal Coordinate Analysis (PCoA) plots from tree species abundance-based Bray-Curtis dissimilarities across nine fungal functional guilds. The richness, Shannon diversity, community composition and abundance of each fungal functional guild were used as explanatory variables.

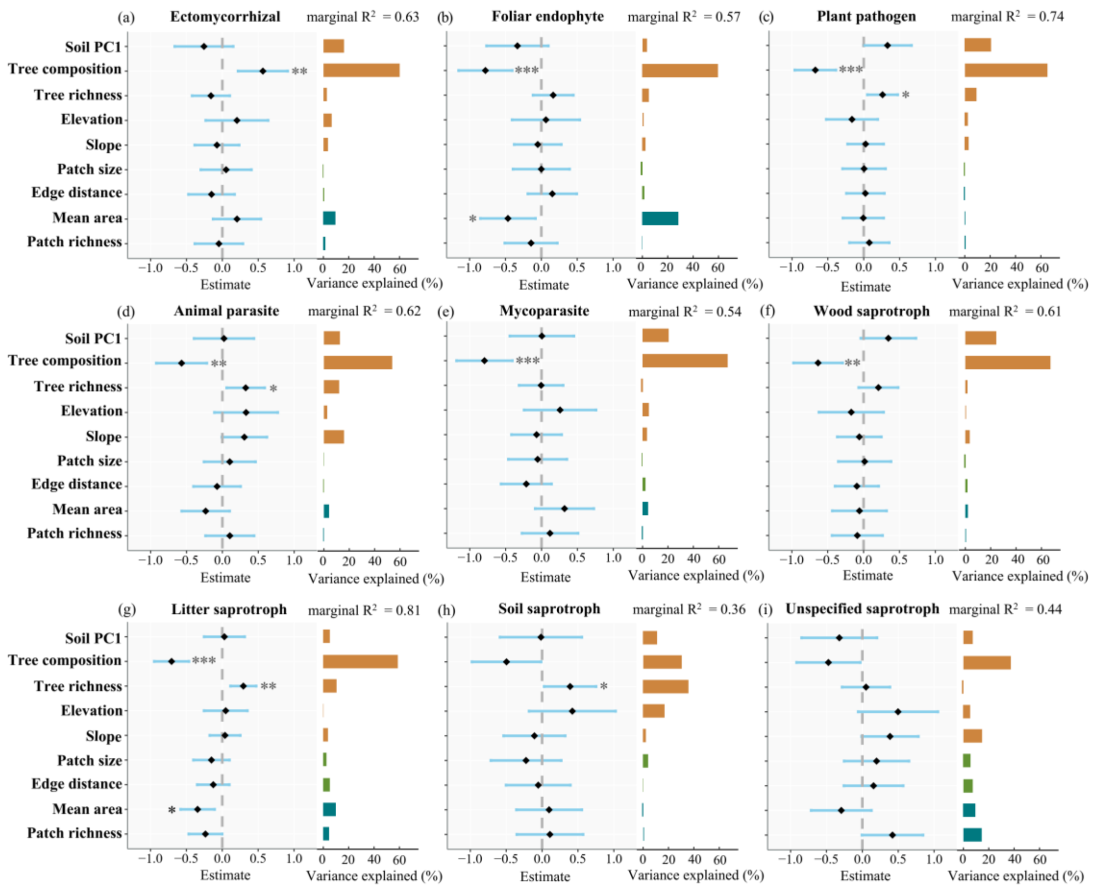

Supplementary Figure 6. Effects and relative contributions of multi-scale environmental factors on soil fungal richness. Panels (a)–(i) show parameter estimates (corresponding 95% confidence intervals) and explained variance of each predictor for different fungal functional guilds from multivariate linear mixed-effects models (LMMs) (n = 30 biologically independent samples). Local factors include soil PC1, tree composition, tree richness, elevation and slope. Patch factors include $\log_{10}$ transformed patch size and edge distance. Landscape factors include $\log_{10}$ transformed mean of patch area (mean area), and patch richness. The significance was tested by *p*-value as *** $p \leq 0.001$; ** $p \leq 0.01$; * $p \leq 0.05$. See Supplementary Data 1 for details and the exact *p*-values.

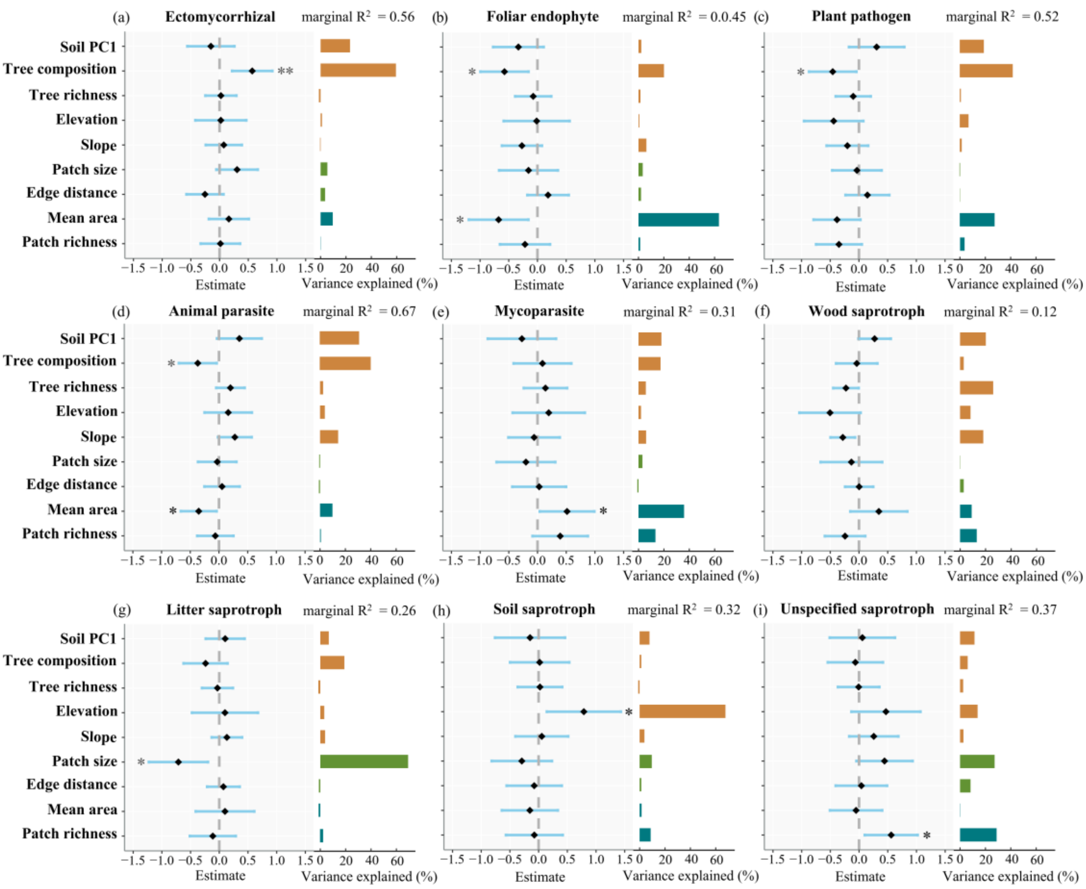

Supplementary Figure 7. Effects and relative contributions of multi-scale environmental factors on soil fungal abundance. Panels (a)–(i) show parameter estimates (corresponding 95% confidence intervals) and explained variance of each predictor for different fungal functional guilds from multivariate linear mixed-effects models (LMMs) (n = 30 biologically independent samples). Local factors include soil PC1, tree composition, tree richness, elevation and slope. Patch factors include $\log_{10}$ transformed patch size and edge distance. Landscape factors include $\log_{10}$ transformed mean of patch area (mean area), and patch richness. The significance was tested by *p*-value as *** $p \leq 0.001$; ** $p \leq 0.01$; * $p \leq 0.05$. See Supplementary Data 1 for details and the exact *p*-values.

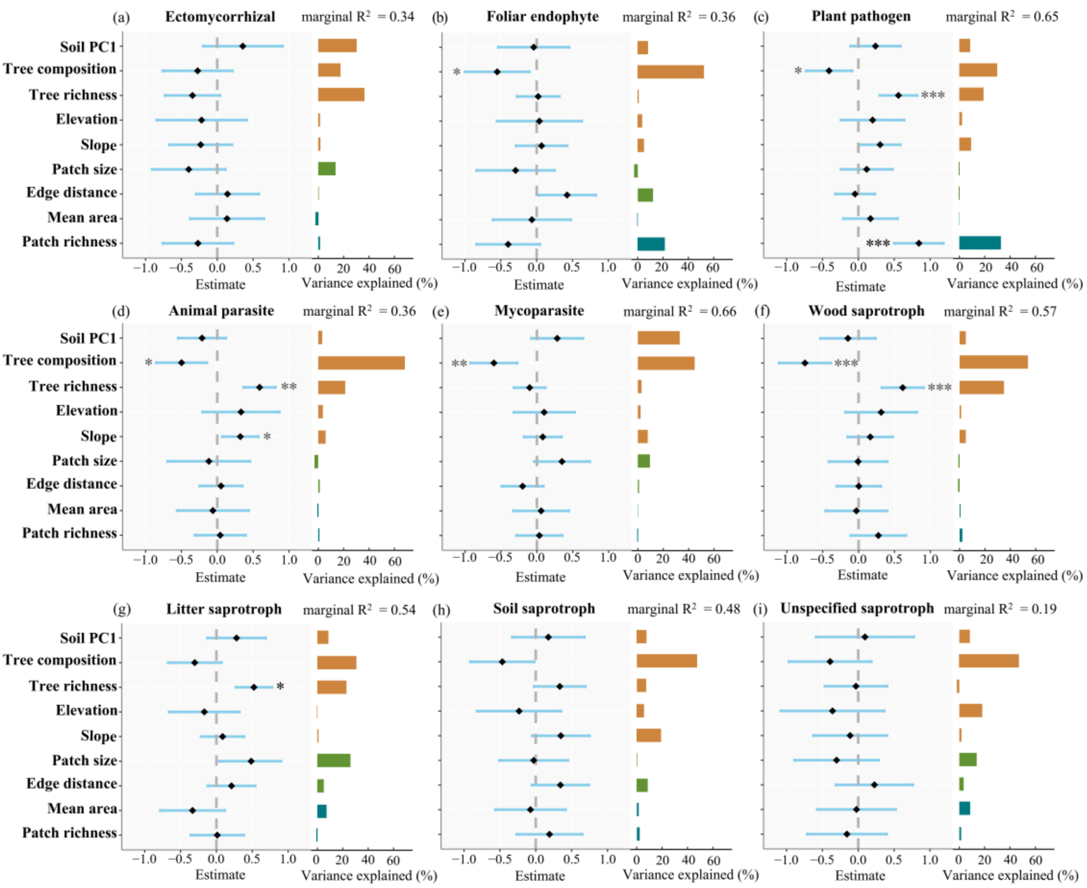

Supplementary Figure 8. Effects and relative contributions of multi-scale environmental factors on soil fungal Shannon diversity. Panels (a)–(i) show parameter estimates (corresponding 95% confidence intervals) and explained variance of each predictor for different fungal functional guilds from multivariate linear mixed-effects models (LMMs) (n = 30 biologically independent samples). Local factors include soil PC1, tree composition, tree richness, elevation and slope. Patch factors include $\log_{10}$ transformed patch size and edge distance. Landscape factors include $\log_{10}$ transformed mean of patch area (mean area), and patch richness. The significance was tested by *p*-value as *** $p \leq 0.001$; ** $p \leq 0.01$; * $p \leq 0.05$. See Supplementary Data 1 for details and the exact *p*-values.

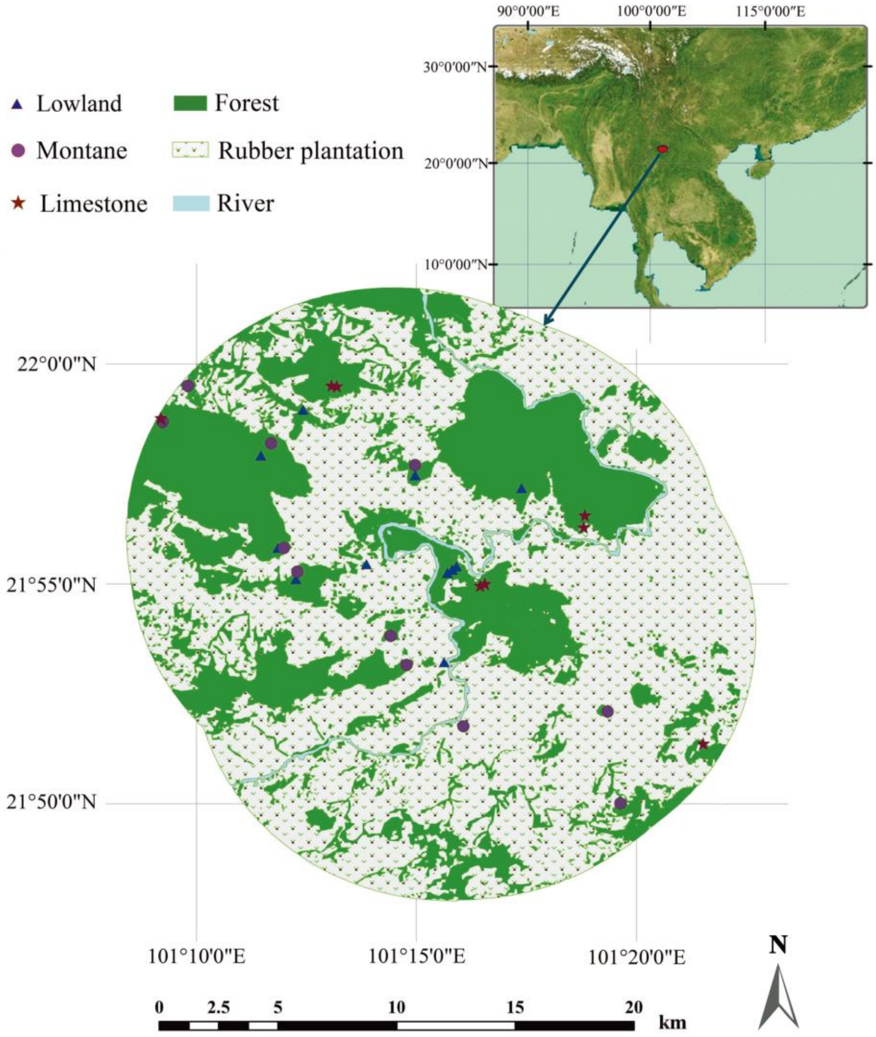

Supplementary Figure 9. Geographical location of the sampling plots in Xishuangbanna. Map showing the locations of 30 sampling plots in Xishuangbanna, southwestern China. Forest types are distinguished by different symbols representing limestone, lowland, and montane forests.

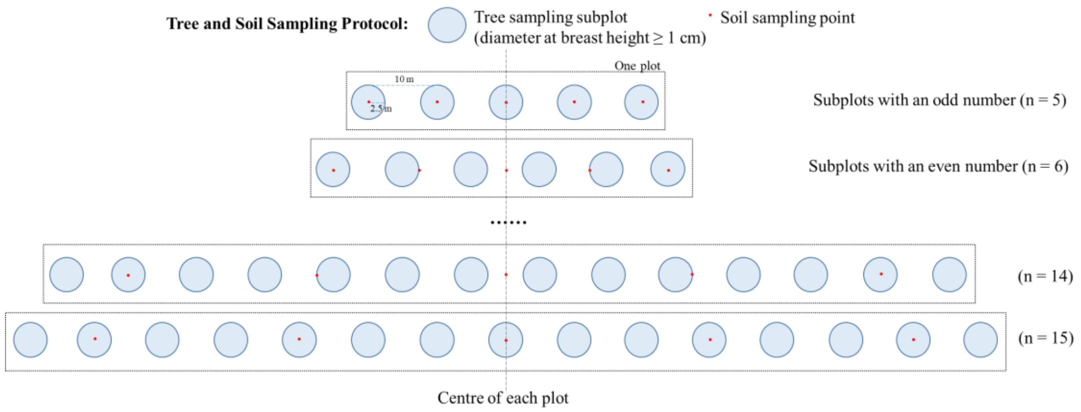

Supplementary Figure 10. Schematic diagram of the tree and soil sampling protocol. Circular subplots with a 5 m diameter (blue circles) were systematically arranged along a transect line within each plot, spaced 10 m apart. The number of subplots per plot varied from 5 to 15 depending on tree density, based on a variable-area sampling method designed to ensure a minimum of 100 trees (DBH ≥ 1 cm) per plot. Soil samples were collected at five evenly spaced points (soil cores with 5 cm in diameter and 10 cm in depth) radiating from the center of each plot, for fungal DNA extraction and soil physicochemical analyses.

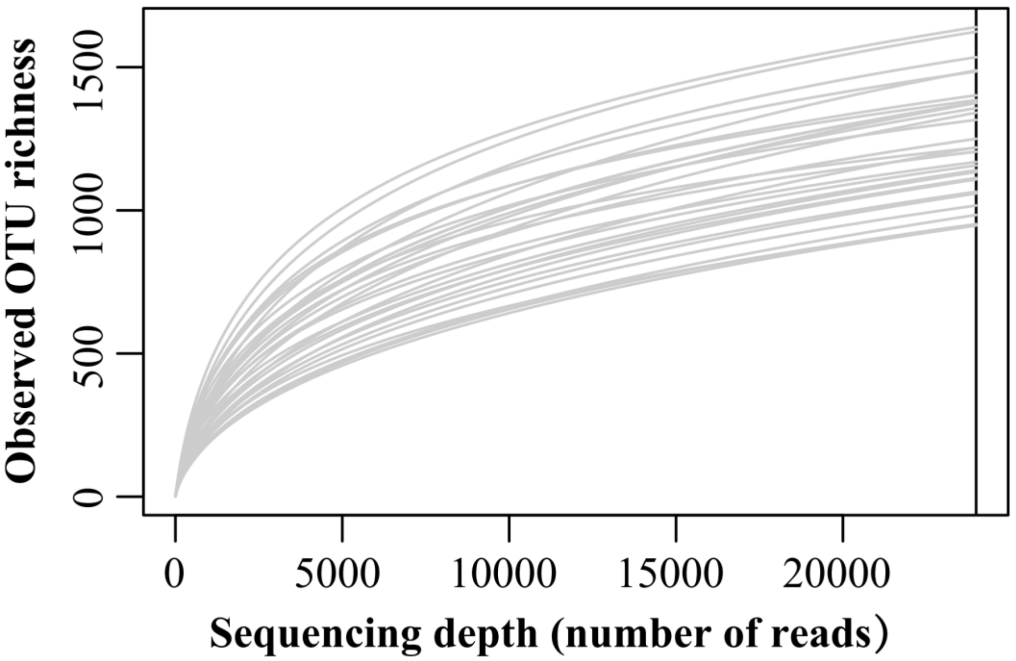

Supplementary Figure 11. Rarefaction curve for fungal sequencing from 30 soil samples. Rarefaction curves show the relationship between sequencing depth and the number of observed operational taxonomic units (OTUs) for each soil sample. Each grey line represents one of the 30 soil samples, indicating that sequencing coverage was sufficient to capture the majority of fungal diversity.
