# Supplementary material for: Cross-scale drivers of soil fungal diversity in fragmented forests of southwestern China
Source: Commun Biol. 2025 Nov 26;8:1689. doi: 10.1038/s42003-025-09091-8 (PMC12658136; doi:10.1038/s42003-025-09091-8)
Supplement: Supplementary file 3 — Description of Additional Supplementary Files [file 42003_2025_9091_MOESM3_ESM.pdf]

Description of Additional Supplementary Files

File name- Supplementary Data 1

File description – Detailed results underlying Figure 4 and Supplementary Figure 6–8 are provided in Supplementary Data 1.
