# Supplementary material for: Cross-scale drivers of soil fungal diversity in fragmented forests of southwestern China
Source: Commun Biol. 2025 Nov 26;8:1689. doi: 10.1038/s42003-025-09091-8 (PMC12658136; doi:10.1038/s42003-025-09091-8)
Supplement: Supplementary file 5 — Reporting Summary [file 42003_2025_9091_MOESM5_ESM.pdf]

nature portfolio

Corresponding author(s): Ying Chen

Last updated by author(s): Oct 16, 2025

Reporting Summary

Nature Portfolio wishes to improve the reproducibility of the work that we publish. This form provides structure for consistency and transparency in reporting. For further information on Nature Portfolio policies, see our Editorial Policies and the Editorial Policy Checklist.

Statistics

For all statistical analyses, confirm that the following items are present in the figure legend, table legend, main text, or Methods section.

n/a	Confirmed	
☐	☒	The exact sample size (*n*) for each experimental group/condition, given as a discrete number and unit of measurement
☐	☒	A statement on whether measurements were taken from distinct samples or whether the same sample was measured repeatedly
☐	☒	The statistical test(s) used AND whether they are one- or two-sided<br>*Only common tests should be described solely by name; describe more complex techniques in the Methods section.*
☐	☒	A description of all covariates tested
☐	☒	A description of any assumptions or corrections, such as tests of normality and adjustment for multiple comparisons
☐	☒	A full description of the statistical parameters including central tendency (e.g. means) or other basic estimates (e.g. regression coefficient) AND variation (e.g. standard deviation) or associated estimates of uncertainty (e.g. confidence intervals)
☐	☒	For null hypothesis testing, the test statistic (e.g. *F*, *t*, *r*) with confidence intervals, effect sizes, degrees of freedom and *P* value noted<br>*Give P values as exact values whenever suitable.*
☒	☐	For Bayesian analysis, information on the choice of priors and Markov chain Monte Carlo settings
☒	☐	For hierarchical and complex designs, identification of the appropriate level for tests and full reporting of outcomes
☐	☒	Estimates of effect sizes (e.g. Cohen's *d*, Pearson's *r*), indicating how they were calculated

Our web collection on statistics for biologists contains articles on many of the points above.

Software and code

Policy information about availability of computer code

Data collection	No software was used to collect data.
Data analysis	Open source code R 4.2.0 is used for statistical analyses (R core team).

For manuscripts utilizing custom algorithms or software that are central to the research but not yet described in published literature, software must be made available to editors and reviewers. We strongly encourage code deposition in a community repository (e.g. GitHub). See the Nature Portfolio guidelines for submitting code & software for further information.

Data

Policy information about availability of data

All manuscripts must include a data availability statement. This statement should provide the following information, where applicable:

- Accession codes, unique identifiers, or web links for publicly available datasets
- A description of any restrictions on data availability
- For clinical datasets or third party data, please ensure that the statement adheres to our policy

Raw sequencing data have been deposited in the NCBI SRA under accession code PRJNA1171718. All other data supporting the findings of this study, including the source data for Figure 1–5 and the taxonomic and functional guild classification files, are available on Figshare at https://doi.org/10.6084/m9.figshare.28642496. Detailed results underlying Figure 4 and Supplementary Figure 6–8 are provided in Supplementary Data 1.

Research involving human participants, their data, or biological material

Policy information about studies with human participants or human data. See also policy information about sex, gender (identity/presentation), and sexual orientation and race, ethnicity and racism.

Reporting on sex and gender	n/a
Reporting on race, ethnicity, or other socially relevant groupings	n/a
Population characteristics	n/a
Recruitment	n/a
Ethics oversight	n/a

Note that full information on the approval of the study protocol must also be provided in the manuscript.

Field-specific reporting

Please select the one below that is the best fit for your research. If you are not sure, read the appropriate sections before making your selection.

☐ Life sciences ☐ Behavioural & social sciences ☒ Ecological, evolutionary & environmental sciences

For a reference copy of the document with all sections, see nature.com/documents/nr-reporting-summary-flat.pdf

Ecological, evolutionary & environmental sciences study design

All studies must disclose on these points even when the disclosure is negative.

Study description	30 survey plots were established to evaluate the consequences of fragmentation on biodiversity in this region from 2007. These plots, spanning 17 patch sizes, were classified into three forest types: limestone, lowland, and montane.
Research sample	In May 2021, we collected soil samples for all the 30 plots and kept stored at -20°C for fungal DNA extraction.
Sampling strategy	Five soil cores (5 cm in diameter and 10 cm in depth) were collected at equal distances radiating outward from the center of each plot.
Data collection	To investigate the impact of local-scale factors on soil fungal diversity, we considered soil properties, tree species diversity of richness, Shannon diversity and community composition, elevation and slope (all collected on site). To assess the impact of patch and landscape factors on soil fungal diversity, we considered patch-scale factors of patch size and edge distance, and landscape-scale factors of mean of patch area (mean area), edge density, patch density, and patch richness within a 2 km spatial radius, using images from the China's Land-Use/Cover Datasets (CLCD, 30 m resolution).
Timing and spatial scale	All the samples were collected in May 2021. The landscape factors were calculated from a land cover map with 30 m resolution from China's Land-Use/Cover Datasets within a 2 km spatial radius.
Data exclusions	No data were excluded from the analyses.
Reproducibility	Three forest types and replicated in Xishuangbanna fragmented forest.
Randomization	In each plot, five samples were collected and pooled into one composite sample.
Blinding	Blinding is not revelant since we performed no artificial control in the experimental design.

Did the study involve field work? ☒ Yes ☐ No

Field work, collection and transport

Field conditions	The field is part of the Indo-Burma biodiversity hotspot. The annual mean temperature of this region is about 21.5 °C and annual mean precipitation about 1,563 mm, with around 80% of the rain falling during the rainy season from May to October
Location	The fragmented forests studied are situated within a 20 km-diameter around the Xishuangbanna Tropical Botanical Garden, Chinese Academy of Sciences, Yunnan Province, southwestern China.

Access & import/export	Samples were collected by authors using local permits.
Disturbance	This study did not cause any environmental disturbance.

Reporting for specific materials, systems and methods

We require information from authors about some types of materials, experimental systems and methods used in many studies. Here, indicate whether each material, system or method listed is relevant to your study. If you are not sure if a list item applies to your research, read the appropriate section before selecting a response.

Materials & experimental systems

n/a	Involved in the study
☒	☐ Antibodies
☒	☐ Eukaryotic cell lines
☒	☐ Palaeontology and archaeology
☒	☐ Animals and other organisms
☒	☐ Clinical data
☒	☐ Dual use research of concern
☒	☐ Plants

Methods

n/a	Involved in the study
☒	☐ ChIP-seq
☒	☐ Flow cytometry
☒	☐ MRI-based neuroimaging

Plants

Seed stocks	n/a
Novel plant genotypes	n/a
Authentication	n/a
